# Supplementary material for: Towards unified AI-driven fracture mechanics: the extended deep energy method (XDEM)
Source: Nat Commun. 2026 Aug 15;17:8492. doi: 10.1038/s41467-026-76748-1 (PMC13484471; doi:10.1038/s41467-026-76748-1)
Supplement: Supplementary file 3 [file 41467_2026_76748_MOESM3_ESM.zip › pic/Results/Crack_propagation/Kinking/crack_kinking.pdf]

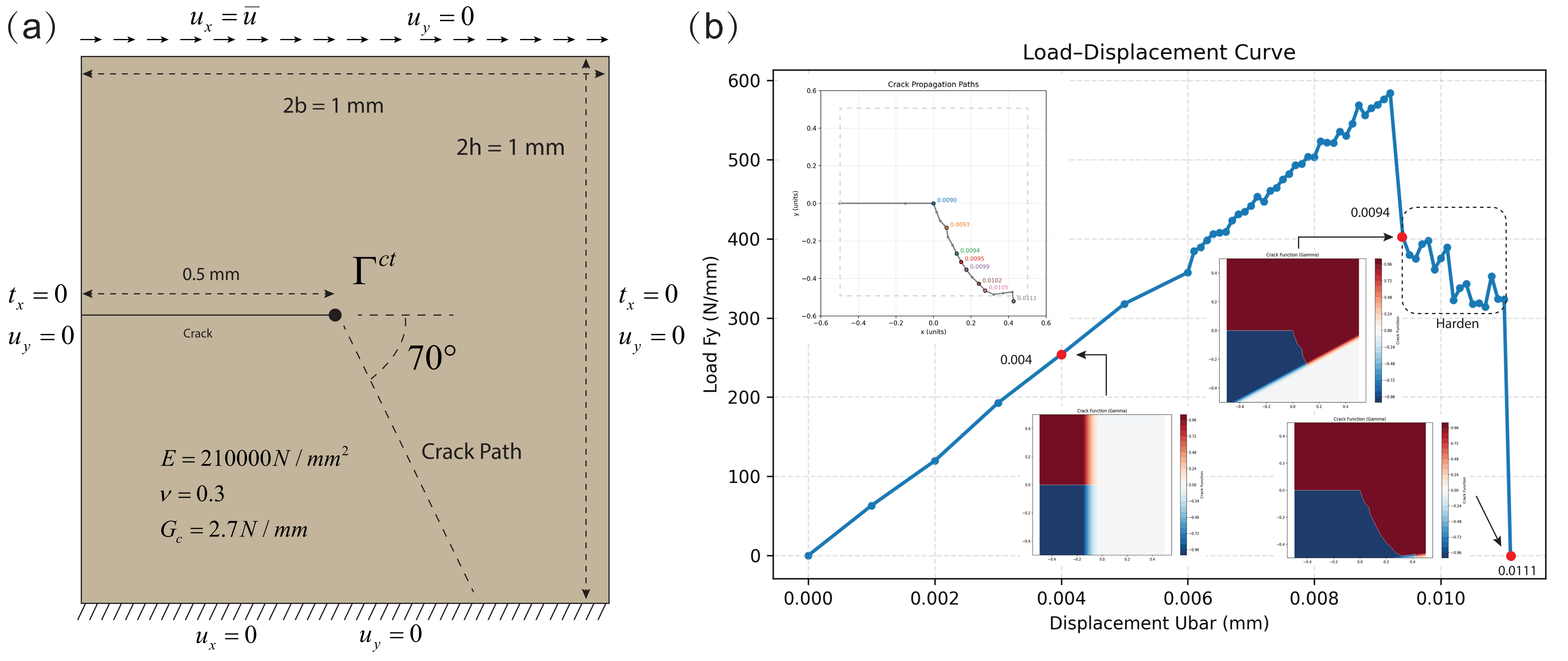

(a)
$u_x = \bar{u}$
$u_y = 0$
2b = 1 mm
2h = 1 mm
0.5 mm
Γ^{ct}
$t_x = 0$
$u_y = 0$
Crack
70°
$t_x = 0$
$u_y = 0$
Crack Path
$E = 210000 N / mm^2$
$\nu = 0.3$
$G_c = 2.7 N / mm$
$u_x = 0$
$u_y = 0$
(b)
Load–Displacement Curve
Load Fy (N/mm)
Displacement Ubar (mm)
Crack Propagation Paths
y (units)
x (units)
0.0090
0.0093
0.0094
0.0095
0.0099
0.0102
0.0105
0.0111
Crack Function (Gamma)
Crack Function
0.004
0.0094
Harden
0.0111
